# Plasmalemmal VDAC-1 corroborated as amyloid Aß-receptor

**DOI:** 10.3389/fnagi.2015.00188

**Published:** 2015-09-30

**Authors:** Friedrich P. Thinnes

**Affiliations:** Max-Planck-Gesellschaft München, GöttingenGermany^†^

**Keywords:** plasmalemmal VDAC-1, amyloid Aß receptor, induced cell death, cell volume regulation, apoptosis, GxxxG motif

## Introduction

Some 40 years ago while spending the night in front of TV and waiting for Neil Armstrong to step down the lander of Apollo 11 to the surface of the moon, indeed, I could not imagine to witness, another event of corresponding relevance for mankind in my lifetime. However, the recent report by Siemers and colleges on slowing down progress of Alzheimer Disease of people showing mild but proven Alzheimer symptoms by Solanezumab antibodies is of that size (Siemers et al., 2015).

## Thesis: Alzheimer disease rests on cell death induction by amyloid Aß mono- and oligomers

**Figure 1:**
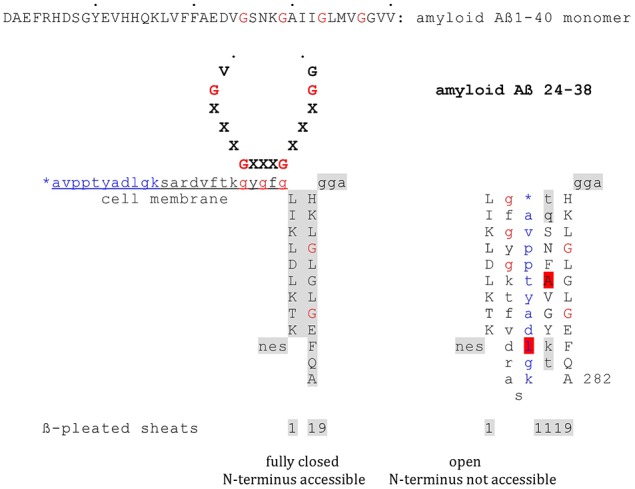
**Model on the putative interaction of plasmalemmal VDAC-1 and Aß monomers via GxxxG motifs**. Highly schematic two-dimensional projections of human type-1VDAC summarizing data from my own lab and the laboratories of Vito De Pinto, Roland Benz, Varda Shoshan-Barmatz, Carmen Mannella, and Jeff Abramson; modified fom Thinnes (2015b). Fully closed state and open state of native VDAC-1 are compared. Noteworthy, the GxxxG motif has been shown to work as an ATP binding site may putatively figure as a peptide interaction/membrane perturbation motif, too.

From my point of view the observations presented by Siemers et al. (2015) not only represent a therapeutic breakthrough. The effects observed also broaden the understanding of the pathogenesis of Alzheimer Disease, this by plainly pointing to induced neuronal cell death as basic in AD. The data thus support a proposal of mine first made in 2010 (Thinnes, 2010, 2011, 2012a,b). For a highly shematic trial to graphically demonstrate the concept see Figure 1.

Accordingly, plasmalemmal VDAC-1 (Swiss Prot. P21796) in situ works as a receptor of amyloid Aß mono- or oligomers. In line, it has been shown that docking of those Aß forms to cell surfaces result in an opening of cell membrane-standing VDAC-1, a process finally ending in neuronal cell death. In consequence, whenever critical brain regions and their redundant structures are affected this way neuronal loss must be expected. In contrary, to neutralize amyloid Aß mono- or oligomers by adequate monoclonal antibodies should minimize Aß toxicity, in other words slow down AD progress (Siemers et al., 2015).

The voltage dependent anion channel (VDAC) is an archaic channel and thus suggested to be involved in housekeeping functions. The channel is well established in the outer mitochondrial membrane, here playing its role in the intrinsic apoptotic pathway (Huang et al., 2015). It is this way of proven relevance for Alzheimer's Dementia (Demetrius et al., 2015).

A first report on an extra-mitochondrial expression of VDAC-1 came up in 1989 showing that human lymphocytes carry a heavy load of the molecule in their plasmalemma. Those data were meanwhile corroborated by studies of several laboratories using different approaches with several cell lines and tissues: e.g., immuno-topochemistry on the light and electron microscopy level, raster electron microscopy, mitochondrial compartment marker controlled cell membrane enrichment, recombinant VDAC1 expression combined with GFP-labeling, voltage-clamp, and patch-clamp studies on *Xenopus* oocytes and mammalian astrocytes, video monitoring of the swelling behavior of HeLa cells; for details see Thinnes and Reymann (1997), Thinnes (2015a,b) and www.futhin.de. A series of recent papers, from my point of view, raises cell membrane-integration of VDAC-1 beyond reasonable doubt, this by connecting plasmalemmal VDAC-1 to cell biologic mechanisms that make the issue lucid (Fernandez-Echevarria et al., 2014; Li et al., 2014; Tewari et al., 2014).

Concerning functions of plasmalemmal VDAC-1, after studies focussed on the regulatory volume decrease (RVD) of HeLa cell (Thinnes et al., 2000) or murine respiratory epithelial cells (Okada et al., 2004), respectively, had shown that cell membrane-integrated type-1 VDAC is part of the cell volume regulatory system of mammalian cells data came up indicating that plasmalemmal VDAC-1 plays its role in apoptosis, too.

In a first effort Elinder et al. (2005) elaborated that opening of VDAC-1 in the plasma membrane precedes the activation of caspases in neuronal apoptosis, here induced by staurosporine. In other words, the authors documented that keeping plasmalemmal type-1 VDAC of neurons closed by different specific antibodies abolishes the apoptotic volume decrease (AVD) of these cells.

Next, endeavors for Raquel Marin's laboratory, Tenerife, corroborated and widened Elinder's data. A first study on the toxic effect of amyloid Aß peptides on septal (SN56) and hippocampal (HT22) neurons, on the one hand, proved another time that blocking VDAC in cell membranes by two different anti-porin antibodies means preventing an apoptotic development of the cells. On the other hand, it showed that VDAC and the estrogen receptor α (mERα) in association with caveolin-1 co-localize and interact in cell membrane caveolae, mERα working toward neuroprotection (Marin et al., 2007). The topographic relationship of the molecules was further specified demonstrating that both are integrated in caveolar lipid rafts (Marin et al., 2008). It has, furthermore, been demonstrated that APP and BACE1 increase interactions in neuronal lipid rafts with progressing AD what may be explained by changes in the physicochemical properties of these microdomains. Indeed, that also induces a further association of, both, amyloid beta aggregates and APP in lipid rafts (Fabelo et al., 2014; Díaz et al., 2015). The group meanwhile presented additional data to demonstrate that the interaction of VDAC and mERα in caveolae from human cortex is altered in Alzheimer disease (Ramírez et al., 2009; Marin, 2011), results which appear to be in line with an early 2D-electrophoresis report on differences in VDAC content of biopsies taken from normal or Alzheimer brains, respectively (Yoo et al., 2001).

Finally, Reddy's laboratory elaborated relevant data on effects of amyloid Aß on VDAC-1, here mostly focussed on mitochondrial processes (Manczak et al., 2011; Manczak and Reddy, 2012; Reddy, 2013).

Asking for a putative mechanism of interactions of cell membrane-standing type-1 VDAC and amyloid mono- and oligomers, it helps to notice that plasmalemmal VDAC-1 carries a critical GxxxG motif cell outside (Thinnes, 2015a), while amyloid Aß40/42 includes several of them in series (Thinnes, 2010, 2011). However, GxxxG motifs are established aggregation and membrane perturbation motifs, furthermore showing some affinity to cholesterol, phenomena broadly discussed in Alzheimer literature (Munter et al., 2010; Gromek et al., 2014). Concerning other VDAC/peptide interactions see Prezma et al. (2013), Shimizu et al. (1999), www.futhin.de.

Given this background recent data on an enhancement of BACE1 expression of hypometabolic neurons (Zhang et al., 2010) made me ask if amyloid Aß, cut from ubiquitous amyloid precursor protein (APP) by ß-secretase BACE1 and γ-secretase, may spot wise induce neuronal cell death via opening ubiquitous VDAC-1 in cell membranes of critical brain regions - a process ending in Alzheimer Disease (Thinnes, 2010, 2011). The authors, remembering cerebral hypometabolism and amyloid accumulation as prevailing neuropathological characteristics of Alzheimer Disease had tried to define effects of neuronal hypoactivity on amyloid plaque pathogenesis in the Tg2576 transgenic mouse model of Alzheimer's disease. They found that unilateral naris-occlusion resulted in an elevation of the ß-secretase BACE1 in neuronal terminals of deprived bulb and piriform cortex in young adult mice (Zhang et al., 2010; Xiao et al., 2015).

## Conclusion

Taking for granted that (1) neuronal cells having lost their balance show enhanced BACE1 expression and thus increased Aß production, (2) amyloid Aß mono- and/or oligomers dock to cell membrane-standing type-1 VDAC of neuronal cells via GxxxG motifs, (3) docking reactions result in plasmalemmal VDAC-1 channel opening followed by a form of extrinsic cell death, and (4) Solanezumab antibodies neutralize Aß oligomers by agonist scavenging, a revised version of the amyloid cascade hypothesis of Alzheimer pathogenesis comes up.

Accordingly, familial as well as sporadic Alzheimer's disease—downstream of APP processing—can be seen as resting on a form of extrinsic induced cell death, this via opening cell membrane-standing VDAC-1 (= receptor). The process is boosted by excessive amyloid Aß (= agonist) production via increased processing of the amyloid precursor protein (APP) of weakening cells of critical brain regions.

However, the synopsis of a series of solid data from several laboratories helps to understand the phenomenology and pathogenesis of either form of AD. Phenotypically mild at the beginning, increasing brain function disturbances evidenced by worsening stages of the disease over time point to a progressive process on the somatic level.

First singular or just a few cells being affected, over time a burden of cell deaths accumulates that finally ends in Alzheimer's Dementia whenever critical brain regions and their redundant structures are affected (Jean et al., 2015). In line, to block free amyloid by specific antibodies allows slowing down Alzheimer's Dementia as recently indicated by the nowadays moon lading of Siemers and colleges.

Finally, the model presented at least formally allows explain the reverse relationship of AD and cancer by pointing to processes (Thinnes, 2012a,b; Chiu et al., 2015; Ganguli, 2015) which may work in parallel to mitochondria related events in Alzheimer pathogenesis as proposed by Demetrius et al. (2015).

However, my hope is that looking on Alzheimer pathogenesis in the context of induced cell death will stimulate the field, this the more as recent literature indicates that amyloid Aß may work this way in other places (Martí-Fàbregas et al., 2014; Kaffashian et al., 2015; Zetterberg, 2015).

While this manuscript was under review a publication appeared that, from my point of view, adds further relevant observations on effects of antibodies on amyloid building. The study presented by Liu et al. (2015) reports on three monoclonal antibody preparations elaborated against different epitopes inside the amyloid Aß peptide. One of those called 6E10 a) *in vitro* disaggregates artificial amyloid fibrils and thus increases the number of Aß oligomers, while b) the injection of co-incubates into the lateral ventricle of 6-month-old C57 mice increased the neurotoxicity *in vivo*.

However, to raise amyloid Aß oligomers increases the risk of their docking to plasmalemmal VDAC-1 finally resulting in the induction of accumulating neuronal cell deaths. From here: To raise amyloid Aß oligomers accelerates AD progress. The authors call the phenomenon dust-raising.

From here, it is tempting to think Alzheimer plaques formation as a salutary form of wipe-the-dust procedure that may even protect from Alzheimer Dementia. In other words: does plaque formation work as a buckler?

### Conflict of interest statement

The author declares that the research was conducted in the absence of any commercial or financial relationships that could be construed as a potential conflict of interest.
